# Worker exposure to persistent organic pollutants, as polybrominated diphenyl ethers, and biological hazards during the processing of waste upholstered domestic seating in Great Britain

**DOI:** 10.1093/annweh/wxaf066

**Published:** 2025-10-23

**Authors:** Rebecca J Gosling, Andrew T Simpson, Claire Bailey, Peter E J Baldwin, Samantha Lord

**Affiliations:** Health - Exposure and Control, Health and Safety Executive (HSE), HSE Science and Research Centre, Harpur Hill, Buxton, Derbyshire SK17 9JN, United Kingdom; Health - Exposure and Control, Health and Safety Executive (HSE), HSE Science and Research Centre, Harpur Hill, Buxton, Derbyshire SK17 9JN, United Kingdom; Health - Exposure and Control, Health and Safety Executive (HSE), HSE Science and Research Centre, Harpur Hill, Buxton, Derbyshire SK17 9JN, United Kingdom; Health - Exposure and Control, Health and Safety Executive (HSE), Wellington Place, 2nd Floor, 7 and 8 Wellington Place, Leeds, West Yorkshire LS1 4AP, United Kingdom; Specialist Division, Health and Safety Executive (HSE), 100 Temple St, Redcliffe, Bristol BS1 6AG, United Kingdom

**Keywords:** control measures, PBDE, persistent organic pollutants, POPs, worker exposure, WUDS

## Abstract

Pressure to increase rates of recycling in Great Britain is expected to increase to meet circular economy and net zero drivers. There are concerns about worker exposure to persistent organic pollutants (POPs) during the processing and recycling of waste upholstered domestic seating (WUDS). The aim of this study was to understand worker exposures to POPs, specifically the flame retardants polybrominated diphenyl ethers (PBDEs), and other airborne substances hazardous to health, when WUDS go through the recycling process. Five WUDS processing sites were visited by a health and safety executive occupational hygienist, who collected worker and static air samples, bulk dust and bulk material samples, and assessed control measures in use. All exposures to inhalable dust and PBDEs were significantly below occupational exposure limits, while exposures to airborne bacteria and fungi were elevated at most sites. Exposures to endotoxins were above the recommended health-based nonbinding occupational exposure limits at 4 sites. Across all sites, recommended control measures were only partially met, indicating that exposures to airborne dust and biological agents could be reduced that these sites.

What's Important About This Paper Processing waste of upholstered domestic furniture may expose recycling workers to hazards in the furniture. This study demonstrates that while exposure to polybrominated diphenyl ether (PBDE) flame retardants is low, recycling workers are exposed to biological hazards, specifically endotoxins. Use of recommended control measures was incomplete at sites visited, providing opportunities for exposure reduction using existing strategies.

## Introduction

Rates of recycling in Great Britain are expected to increase over coming years to meet circular economy and net zero drivers, and to reduce use of landfill and the export of waste to developing countries (Nandy et al. 2022). The recycling process creates a risk of workers being exposed to hazardous substances previously applied to the items being recycled. One such group of substances is brominated flame retardants (BFR). Some BFRs, including polybrominated diphenyl ethers (PBDEs), are classified as persistent organic pollutants (POPs). From 1 January 2023, new guidance from the Environment Agency (EA) came into effect regarding the storage and disposal of waste upholstered domestic seating (WUDS) containing POPs (EA 2023). This guidance applied to England, but similar changes were made in Wales and Scotland. As a result, waste is no longer being disposed of in landfill but is required to go through high temperature incineration (Anon 2019; EA 2022).

Incinerating WUDS requires preparatory processing of the waste by shredding it into small pieces. The shredded waste may be blended with other high calorific value shredded waste before being conveyed to an incinerator either baled or loose. This process has the potential to expose workers to PBDEs and other hazardous substances in the dust created during handling and shredding. Exposed workers include drivers operating plant vehicles to load the shredder with WUDS or to load lorries for dispatch of shredded waste, but workers who marshal visiting lorries, perform maintenance tasks or clean the equipment and facility may also be exposed.

Due to their toxicity and persistence, several families of POPs, including PBDEs, have been listed in the 2001 Stockholm Convention to protect human health and the environment (Statutory Instruments 2007; Anon 2019). While the Stockholm Convention led to the reduction of POPs in consumer products, they have persisted in legacy materials, and concerns remain around exposures associated with the processing and recycling of legacy waste materials which have historically been treated with PBDEs (Keeley-Lopez et al. 2021).

The term PBDE covers a range of 209 compounds (congeners) (ATSDR 2017) that were marketed as 3 mixtures: commercial pentabromodiphenyl ether (c-PentaBDE), commercial octabromodiphenyl ether (c-OctaBDE), and commercial decabromodiphenyl ether (c-DecaBDE). These mixtures are wide-ranging and variable mixtures of PBDE congeners. However as a guide, c-PentaBDE generally contains tri- to hexa-BDE homologs, c-OctaBDE contains hexa- to deca-BDE, and c-DecaBDE contains octa- to deca-BDE (ATSDR 2017). Some PBDEs release bromine atoms when exposed to heat, which prevents the BFR-coated materials from reaching their ignition temperature and minimizes the spread of fire (Altarawneh et al. 2018). In some cases, synergists such as antimony trioxide are added to increase the fire retardation properties of PBDEs, particularly deca-BDE. This combination was widely used in textiles, and therefore the presence of bromine coupled with antimony provides a good indication of the presence of deca-BDE. Where bromine is present without antimony trioxide there may be other BFRs (eg hexabromocyclododecane and tetrabromobisphenol A) or other brominated compounds present (Keeley-Lopez et al. 2021).

The most common sources and pathways of human exposure to PBDEs have been reported as dust and indoor air (US-EPA 2010; EFSA Panel on Contaminants in the Food Chain (CONTAM) et al. 2024). Long-term exposure to some POPs compounds and scenarios have been reported to have negative effects on human health including increased cancer risk, reproductive disorders, alteration of the immune system, neurobehavioral impairment, endocrine disruption, genotoxicity, and increased birth defects (WHO 2020). The POPs Review Committee Reports following the Stockholm Convention found that, as a result of long-range environmental transport (and demonstrated toxicity in a range of nonhuman species for penta-BDE), commercial PBDEs were likely to cause significant adverse effects on human health (UN 2006, 2007, 2014). Studies have reviewed occupational exposure to POPs, including PBDEs (Sjödin et al. 2003; Hebisch and Linsel 2012). Exposure was highest to BDE-153, a hexa-BDE congener, among workers dismantling electronics, a significant data gap was identified regarding exposure to BDE-209 (DecaBDE) that has not been addressed in recent research (Sjödin et al. 2003). This review, however, did not include exposures from WUDS.

There is very limited data on worker exposure for PBDEs and there are no workplace exposure limits (WELs) within Great Britain. Exposure limits have been proposed by others, depending on composition, although none of these are legally binding. The worker occupational exposure limit for commercial PentaBDE has been proposed at 0.7 mg/m^3^, as an 8-h time weighted average (TWA; IFA no date). For OctaBDE, the limits range from 0.1 to 0.2 mg/m^3^ 8-h TWA (IFA no date; SCOEL 2012), depending on whether the commercial mixture or the homologs are being considered. For commercial DecaBDE, the proposed exposure limit is 5 mg/m^3^ 8-h TWA (TERA no date).

Along with chemical hazards, WUDS may also contain microbiological hazards. The Health and Safety Executive (HSE) have previously reported that workers in municipal waste handling sites in Great Britain were exposed to either low (<1 × 10^4^ Colony Forming Units, CFU/m^3^) or medium (1 × 10^4^ to 1 × 10^6^ CFU/m^3^) levels of bacteria (48% and 44%, respectively), however fungal exposure was most often (69%) at the medium level (HSE 2019). In response to this risk, and to assist with the assessment of workplace bioaerosol exposure the Waste Industry Safety and Health Forum (WISH) published research-based exposure guidelines, produced in conjunction with the HSE, for levels of workplace exposure to bioaerosols in the waste and recycling sector (WISH 2023). Further, endotoxin exposure levels were generally low among workers in municipal waste handling sites in Great Britain, with 63% of samples returning values of <45 EU/m^3^ (HSE 2019). However, 26% of the same samples exceeded the DECOS exposure limit of 90 EU/m^3^ demonstrating the risk of exposure for workers within this industry can be wide-ranging (HSE 2019).

The aim of this study was to understand the risk of worker exposure to POPs used as flame retardants in upholstered furniture and other airborne substances hazardous to health when WUDS are handled in the incineration process. The study was designed to gather initial information about the industry, and may not be representative of the whole sector.

## Materials and methods

Sites were selected for inclusion through discussions with the waste and recycling industry in Great Britain, and companies processing WUDS that expressed a willingness to participate. Occupational hygiene assessments were carried out on 5 sites that regularly process WUDS across England. Visits took place between 1 November 2023 and 15 December 2023. Occupational hygienists carried out exposure monitoring for inhalable dust and bioaerosol in accordance with HSE Guidance MDHS14/4 (HSE 2014) using Institute of Occupational Medicine samplers loaded with glass fiber filters and pumps operating at 2 L/min.

In total, 16 workers across the 5 sites were each fitted with 2 personal air samplers for the duration of their shift. A mean of 879 L of air was sampled for each worker (range 568 to 1,025 L).

And, a total of 18 static background air samples were collected across the 5 sites. A mean of 780 L of air was sampled for each static sampling position (range 581 to 964 L). Sampler locations included points relatively close to shredders, conveyors, and lorry trailer shredded waste loading areas, as well as locations away from sources of airborne dust. These varied by site but were generally within the processing building. Only 2 samples were collected from immediately outside the processing building.

Two sites processed only WUDS (1 at a waste transfer station), 2 processed WUDS alongside domestic municipal waste, and 1 processed WUDS and “nonhazardous bulky waste.” Bulk samples of fabric from WUDS (10 × 10 cm) and settled dust from surfaces within the processing areas, were also taken. The dusts from each site were combined for PBDE analysis. Exposure control measures were observed to determine their effectiveness in preventing or reducing exposures. Management controls such as risk assessments were not specifically assessed.

Gravimetric analysis of inhalable dust samples was performed following HSE Guidance MDHS14/4 (HSE 2014). PBDE analysis was performed on air filters and bulk dust samples using an extraction in a mixture of hexane and acetone, a method adapted from (Fernandes et al. 2004) and gas chromatography with high resolution mass spectrometry. The panel of PBDEs included congeners with IUPAC numbers 17, 28/33 (BDE-28 coelutes with BDE-33), 47, 49*, 66, 71*, 77*, 85, 99, 100, 119*, 126*, 138, 153, 154, 183, and 209. Asterisked congeners were only analyzed for air filter samples. The full analytical method can be found in the Supplementary material. Some extracts contained high levels of BDE-209, which saturated the detector. When the samples were reanalyzed after dilution, accurate quantitation of the control standard was not possible, therefore these results were not include in the analysis. For reporting purposes, PBDEs have been grouped by commercial mixtures, c-PentaBDE, c-OctaBDE, and c-DecaBDE as defined by ATSDR (2017). The EU Scientific Committee on Occupational Exposure Limits (SCOEL) recommended limit for commercial OctaBDE of 0.2 mg/m^3^ was adopted for comparison with both octa- and penta-BDE because of greater assurance as to its derivation based upon the information available (SCOEL 2012). For commercial DecaBDE, the AIHA Workplace Environmental Exposure Level of 5 mg/m^3^ was adopted (TERA no date).

The X-ray Fluorescence (XRF) technique can be used to screen BFR containing samples because the bromine atom is measurable as a marker element (Keeley-Lopez et al. 2021). Fabric samples and bulk dust samples were analyzed as received and screened for the presence of antimony and bromine by energy dispersive XRF spectrometry.

The samples were stored at outdoor ambient temperature for <24 h during transit and then refrigerated until analysis within 3 days. Samples were extracted by aseptically removing filters from the cassettes, placing into 10 mL sterile endotoxin-free phosphate buffered saline (PBS) solution and mixing vigorously for 1 min. Samples were then placed on a roller for 2 h. Bulk dust samples (1 g) were diluted in 10 mL endotoxin-free PBS and mixed vigorously for 1 min. For the mixed processed waste, 10 g was diluted in 100 mL PBS.

For total bacterial and fungal counts 10-fold serial dilutions were made in PBS. 0.1 mL of each dilution (neat concentration to 10^−6^ dilution) was spread in duplicate onto malt agar (Millipore) to measure total fungi, nutrient agar (Oxoid) to measure total bacteria, MacConkey (Oxoid) to measure coliforms, R8 agar (Corry et al. 2011) to measure thermophilic bacteria including actinomycetes. Plates were incubated at the following temperatures: malt at 25 °C and 40 °C; nutrient at 25 °C and 37 °C; MacConkey at 37 °C; and R8 at 55 °C, for 7 days. Samples were categorized using the WISH levels for workplace bioaerosols in the waste and recycling sector (Table 1).

**Table 1.** HSE guidelines published by WISH for levels of workplace bioaerosols in the waste and recycling sector (WISH 2023).

	Total bacteria/total fungi	Aspergillus fumigatus and actinomycetes
Low	<10^3^ CFU/m^3^	—
Medium	10^4^ to 10^5^ CFU/m^3^	>1 × 10^3^ CFU/m^3^
High	>10^6^ CFU/m^3^	>1 × 10^5^ CFU/m^3^

For endotoxin analysis a 1.5 mL subsample of the extracted/diluted dust samples was centrifuged at 1,000 × *g* for 10 min. Dilutions of the supernatant were analyzed using the Kinetic-QCL automated system (Bio-Whittaker Inc., Walkersville, Maryland, USA). Each sample was analyzed with negative and positive controls. The guidance value of 90 endotoxin units (EU)/m^3^ based on personal inhalable dust exposure measured as an 8-h TWA (ACGIH, no date; Health Council of the Netherlands 2010) was adopted.

An unpaired Student *t*-test was used to compare PBDE results (Microsoft® Excel® for Microsoft 365 MSO (Version 2408 Build 16.0.17928.20512) 32-bit). Statistical significance was accepted where *P* < 0.05.

Ethical approval for this project was granted by HSE Research Ethics Panel, submission REP23-007 on 6 October 2023.

## Results

**Fig. 1.**
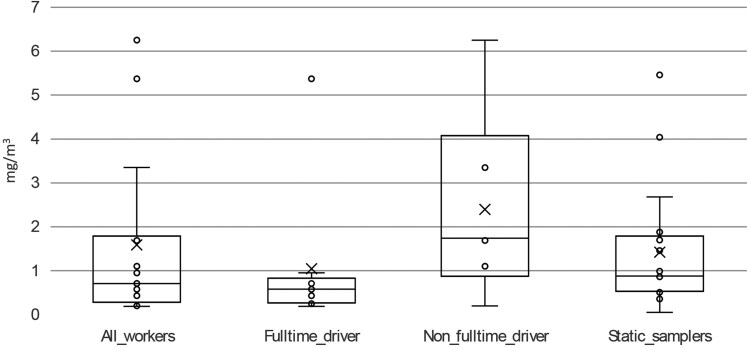
Box-plot of inhalable dust from personal air samplers (*n* = 15), further divided into full-time drivers (*n* = 9) and nonfull-time drivers (*n* = 6), and static air samplers (*n* = 17). The box depicts the quartiles, with mean value indicated by “x” and values “o.”

A full breakdown of results by site and sample type can be found in Table S1. Inhalable dust exposures are presented in Fig. 1. Data for workers identified as full-time drivers has been presented separately to demonstrate differences in exposure.

**Fig. 2.**
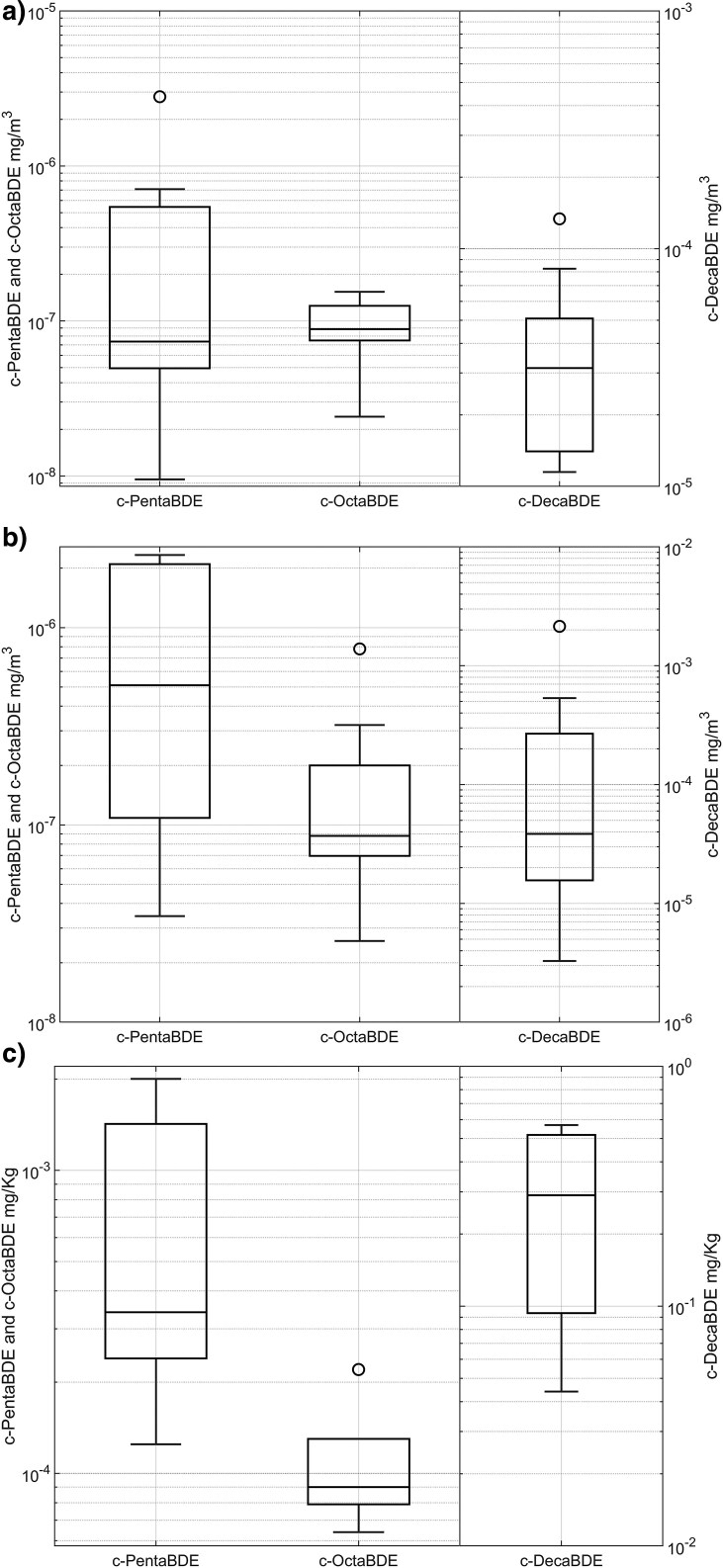
Box-plot of PBDEs concentrations in personal air samples (a; *n* = 15, c-DecaBDE *n* = 11), static air samplers (b; *n* = 17, c-DecaBDE *n* = 14), and dust (c: *n* = 5). Minimum values are approximate values, complicated by presence of values less than the limit of detection. The box depicts the quartiles, with mean value indicated by “x” and values “o.”

The individual PBDE congener exposures have been combined to produce an estimate of exposure to the 3 commercial mixtures, c-PentaBDE, c-OctaBDE, and c-DecaBDE (Fig. 2a–c). No statistically significant difference was observed between the personal and static samples (*P* > 0.05 for each commercial mixture). The relative distribution of congeners quantified in the airborne dust was comparable to that in the bulk settled dust samples.

Twenty-three fabric samples and 14 bulk dust samples were taken over the 5 sites and analyzed for bromine and antimony by XRF (Table 2). All of the fabric samples were found to contain bromine, and 20 contained antimony. All of the bulk dust samples contained bromine, and 11 contained antimony.

**Table 2.** Bromine and antimony presence for fabric and bulk dust samples.

	Fabric (*n* = 23)	Dust (*n* = 14)
Antimony	20	11
Bromine	23	14

**Fig. 3.**
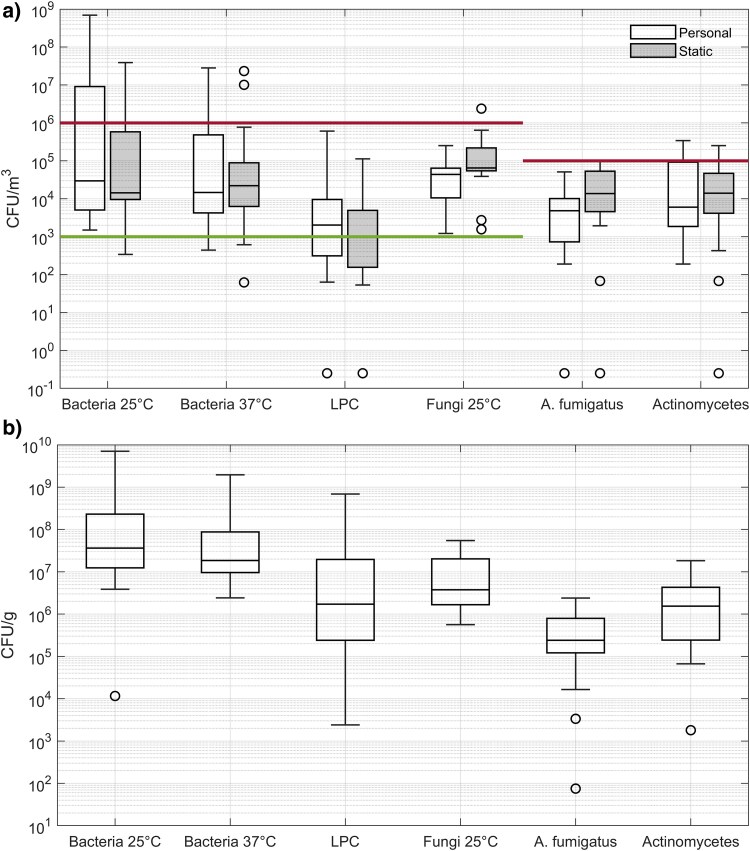
Box-plot of bacterial and fungal counts from personal (*n* = 16) and static air samplers (*n* = 18; a) and dust (*n* = 19; b). The box depicts the quartiles, with mean value indicated by “x” and values “o.” For panel a, the upper horizontal line denotes high levels for total bacteria/fungi (≥1 × 10^6^ CFU/m^3^) and Aspergillus fumigatus/Actinomycetes (>1 × 10^5^ CFU/m^3^) per HSE categorization, while the lower horizontal line denotes low levels for total bacteria/fungi (<1 × 10^3^ CFU/m^3^), no low level values for Aspergillus fumigatus/Actinomycetes are available. LPC, lactase positive coliforms.

**Fig. 4.**
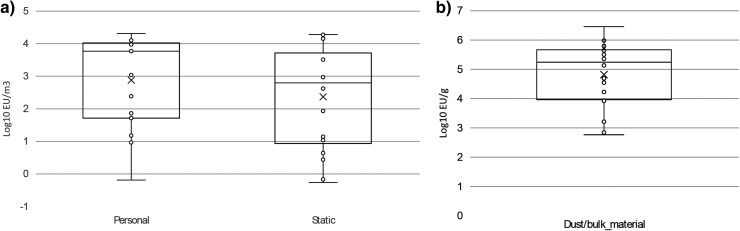
Box-plot of endotoxin concentrations in (a) personal air (*n* = 15) and static samplers (*n* = 183) and in (b) dust/debris and bulk material (*n* = 18). The box depicts the quartiles, with mean value indicated by “x” and values “o.”

The majority of the personal and static air samplers returned bacterial and fungal counts within the WISH guidance medium range (Fig. 3). For the purposes of this study, endotoxin results over the Dutch and ACGIH health-based 8-h TWA limit of 90 EU/m^3^ are considered high (Fig. 4).

At all the sites, work shredding WUDS was confined to within the processing building and access was restricted to site staff and visiting drivers. A summary of the infrastructure and controls by site can be found in Table S2. Many workers benefited from working within a vehicle cab. The vehicles generally had cab air filtration and were operated with the windows closed; however, there was often uncertainty over whether the filters were high efficiency particulate air (HEPA) filters. On some sites, the shredder could be controlled remotely from the vehicle cab. Nevertheless, some activities such as cleaning and maintenance required work outside the cab.

Shredding was performed on machines with open hopper feeds and outlets with no hoods or curtains. Three sites had extensive open conveyor systems with drop transfer points with little enclosure, and 2 sites used smaller more compact mobile shredders.

Ventilation configurations varied among the sites. While none of the sites had local exhaust ventilation (LEV), 3 sites had mechanical general ventilation for the processing building. Two of these sites with mechanical general ventilation sites had roof fans and wall fans, but they were not in use, while a third site had a ducted extract system which was located at ceiling level. Two sites had a high level of natural ventilation in the form of large wall vents with some in relatively close proximity to area where the WUDS were processed. The distribution of the air vents may have allowed some degree of cross-ventilation of the building; however, the vents were reported to be present to minimize condensation. Natural ventilation was also provided by large open vehicular doorways, although at one site automated doorways remained closed unless vehicle access was required. Examination with a dust lamp revealed background airborne dust throughout most of the processing buildings, including areas relatively remote from the processing areas.

The degree of water suppression in use varied over the 5 sites. One site had 2 water dust suppression canons in the WUDS shredding area. Another had an odor and dust absorption/suppression system installed by an air vent. Two others had water mist fans used for both dust and odor suppression positioned above the processing equipment. One mist fan provided comprehensive coverage; the other was not in operation of the day of the visit. The fifth site had ceiling level water mist fans, none of which were in operation. The misters used aqueous solutions containing nonhazardous additives such as surfactants. One contained calcium lignosulfonate that reportedly has binding properties, which may reduce further airborne emissions.

Personal protective equipment required for work in the processing building was largely safety related: high-visibility jackets, hard hats, safety footwear, and eye protection. Two sites issued proximity alarms, which could be fitted to hard hats and gave warning of approaching vehicles. More relevant to the biological and chemical hazard was the protective clothing and respiratory protective equipment (RPE). The workers generally wore workwear, but some wore coveralls. All the sites provided gloves, and at 3 sites they were expected to be worn. RPE was generally deemed unnecessary unless the work was particularly dusty (eg cleaning), but at 1 site RPE was mandatory during operation whenever staff were outside of vehicle cabs. Four sites provided RPE in the form of tight-fitting respirators (usually FFP3 dust masks), and 3 sites had provided face-fit testing to workers. Ear-loop FFP2 masks were used at 1 site, but these are not approved for use by HSE. A powered air purifying respirator (PAPR) had recently been obtained at 1 site. RPE was observed in use at 2 sites during cleaning operations, but at 1 site all 3 workers wore FFP3 respirators with beards. At a third site, 2 workers in surgical masks were observed dry brushing. Only 1 worker was observed wearing appropriate RPE correctly, and he wore a tight-fitting FFP3 mask for his full shift.

All the sites had brushes and shovels available for cleaning. One site also had rakes and scrapers, but 2 sites also had vacuum cleaners. One site employed laborers to clean up during the shift; others stopped processing to clear up periodically or at the end of the shift. One site underwent a thorough clean at weekends and so was not witnessed during the site visit.

## Discussion

This work aimed to provide initial information on the presence of PBDEs and bioaerosol hazards within the WUDS working environment today, and was not designed to characterize the whole WUDS processing sector. It is estimated there are around 66 WUDS processing sites in England (personal communication) and this study included 5 sites from 1 very large and 1 medium sized WUDS processing company within Great Britain. These sites were identified by the companies, and were expected to be the better performers within the industry. Further, exposures measured may vary depending on the type and volume of WUDS being processed.

The inhalable dust exposure samples ranged from 0.19 to 6.25 mg/m^3^, and were below 10 mg/m^3^, the level defined in the COSHH regulations as posting hazard to health as an 8-h TWA (HSE 2013). Given the presence of bioaerosol, however, 10 mg/m^3^ is not an appropriate level of inhalable dust exposure against which to assess respiratory risk and the adequacy of control measures in this setting (HSE 2019). The highest exposure to inhalable dust (6.25 mg/m^3^) was for a baler operator. It was not possible to distinguish the exposures of full-time drivers from other workers, because individual site conditions and exposure during cleaning activities obscured the exposure reduction provided by air filtered cabs. In a prior study by HSE (2019) at a range of municipal waste handling site types (deemed to be employing reasonably good health and safety risk management practices), the 8-h TWA inhalable dust exposures of 8 drivers ranged from 0.22 to 0.95 mg/m^3^, with a median value of 0.39 mg/m^3^ (HSE 2019), comparatively lower than the results reported herein.

Thirteen of the sixteen workers monitored were exposed to measurable quantities of PBDEs, however at concentrations several orders of magnitude below applicable exposure limits. C-decaBDE (principally BDE-209) was the most abundant PBDE, but the highest 8-h TWA exposure of 1.34 × 10^−4^ mg/m^3^ was only 0.003% of the AIHA exposure limit value of 5 mg/m^3^. The exposures to c-PentaBDE were even lower: the highest 8-h TWA exposure of 4.6 × 10^−7^ mg/m^3^ was 0.0000023% of the SCOEL limit value of 0.2 mg/m^3^ for c-OctaBDE, adopted here for comparison. Congeners from all 3 commercial PBDE mixtures were detected in the bulk settled dust. These results are consistent with other work that found c-DecaBDE was consistently present in much larger concentrations than other PBDEs in furniture covers, foam and linings (Keeley-Lopez et al. 2021).

A recent systemic review of occupational exposure to organic flame retardants found no data for exposure to PBDEs during recycling WUDS (Gravel et al. 2019). The review reported worker exposures in e-waste recycling, including a mean of 2.2 × 10^−3^ mg/m^3^ BDE-209 (in c-DecaBDE), 1.9 × 10^−5^ mg/m^3^ BDE-183 (representing c-OctaBDE) and 3.4 × 10^−4^ mg/m^3^ BDE-47 (representing c-PentaBDE). These values are substantially higher than those found in the present study, demonstrating a higher risk of exposure for those recycling e-waste compared to WUDS workers.

At the present time, there is no WELs for microorganisms. Based on HSE work, expert consensus, and published data from Europe and the USA, it is possible to apply banding to workplace bioaerosol exposures as low, medium, or high despite limited dose-response information (WISH 2023). For the majority of workers, airborne bacterial and fungal exposures were low to medium following the WISH guidance. These values are comparable to those reported from municipal waste handling and recycling sites in GB and across Europe (Swan et al. 2003; HSE 2019; Madsen et al. 2021; Hansen et al. 2024; Keen et al. 2024), but higher than those reported in Danish biowaste plants (Madsen et al. 2024). Duquenne et al. (2024) reported lower total bacterial and fungal counts during winter months compared to summer months. The current site visits were all undertaken during winter months, and an increase in exposure may occur during the summer. One site had high levels of airborne bacteria which was attributed to high emissions (including from a lengthy period of cleaning) and limited water mist suppression and general ventilation. Endotoxin levels for 10 out of 15 samples were above the guidance level, indicating the potential to cause a significant health risk to workers; this is also comparable to the results reported from municipal waste handling and recycling sites (Swan et al. 2003; HSE 2019; Madsen et al. 2021; Hansen et al. 2024) but higher than those reported in Danish biowaste plants (Madsen et al. 2024). The levels of bacteria, fungi and endotoxin found within the bulk samples were all high and would present a significant exposure risk to workers should these organisms become airborne or ingested. It is worth noting that 3 of the sites also processed other waste eg municipal waste, using the same shredder, which could therefore be a source of PBDEs and bioaerosols.

Control strategies should be devised following the hierarchy of control and implementing the 8 principles of good practice for the control of exposure to substances hazardous to health, that are included in Regulation 7 and Schedule 2A of the COSHH Regulations (HSE 2013). There is no specific guidance on control of occupational exposure to POPs in the waste and recycling industry, but the EA has produced a regulatory position statement (RPS264) on the shredding of WUDS containing POPs which contains guidance on controls to minimize fugitive dust emissions (EA 2022), and more general guidance on airborne dust exposure control is relevant for reducing POPs exposure. WISH have produced guidance on bioaerosols in waste and recycling (WISH 2023), which includes a table detailing the hierarchy of control and examples of suitable exposure control measures.

The focus in this industry should be on engineering controls, as there were few instances of dust emission control at the source, such as through LEV and enclosures. The shredder feed would be difficult to fully enclose while maintaining access for delivery of bulky WUDS into the hopper, but improvements could be made to conveyors, balers and drop transfer points. However, there were also emissions from delivery of WUDS (tipping), loading of lorry containers with loose shredded waste and during cleaning.

Both the EA and WISH guidance recommend the use of water misting systems to control dust emissions, but there was only limited use of water misters on the sites visited. The remote high-level mist fans may have reduced background levels of airborne dust (ie airborne dust suppression) but would likely only dampen the upper surface of storage piles of unprocessed waste (for airborne dust prevention). During energetic processes, like shredding and tipping, they were observed to be insufficient to prevent emissions. Targeted water misters around the shredder would likely be more effective, but use of excess water may be incompatible with the machine and due to the high levels of bacteria and fungi present may pose an additional health risk.

Mist droplet size influences its effectiveness in controlling dust (NIOSH 2019). Mist similar to light rain is preferable when wetting bulk material for airborne dust prevention, but for effective background airborne dust suppression, the water droplets should be comparable to a fog. No analysis was made of mist droplet size during this study.

The WISH guidance, which is not specific to the shredding of WUDS, recommends extract ventilation of processing buildings to increase air changes. Only one site had extract ventilation but 2 others had good natural ventilation; however, the degree of effectiveness would be weather dependent.

Vehicle cabs provided protection for much of the work, but require that contaminated air is kept out, ie use of appropriate HEPA filters, doors and windows are kept shut and have good seals, and the interior is kept clean (HSE 2018).

The WISH guidance suggests that if exposure cannot be adequately controlled by other means, then RPE should be provided as a last resort and for high exposures should be mandatory. However the provision and use of RPE, and by extension staff training and supervision, across the sites visited required improvement. The RPE supplied was often disposable FFP3 dust masks (with APF of 20), which is the standard given in the WISH guidance. Ear loop RPE was observed at 1 site, though HSE does not recommend using respirators, which rely on ear loops as they do not provide adequate tension to produce a good seal between the mask and the face (HSE 2022). Tight-fitting respirators require wearers to be face fit tested and to be clean-shaven in the area of the face seal (HSE 2013). The 1 worker wearing his FFP3 respirator correctly wore it for the whole shift, though this type of respirator is designed for periods of short-term use—eg, <1 h (HSE 2013). PAPRs would be more appropriate for long-term use and the site was reportedly in the process of acquiring 1.

There was limited use of vacuums for cleaning, but dry brushing appeared to be common. Brushes have the potential to produce significant airborne dust emissions. A scraper, used with care, may produce less airborne dust than dry brushing, but vacuum cleaner would greatly reduce exposure during the removal of settled dust.

## Conclusions

All exposures to inhalable dust were below the 10 mg/m^3^ value at which any dust is defined as hazardous to health in the COSHH Regulations. While this would suggest dust is being controlled, the elevated levels of bioaerosols recorded indicate that more could be done to reduce worker exposure to dust-related hazards. Exposure to endotoxin was above the Dutch and ACGIH recommended health based occupational exposure limit at 4 of the 5 sites, again highlighting the potential risk to health from airborne dust, and the opportunity for improved control measures to be implemented. Across the sites visited, observed control measures were not fully utilized, and the data demonstrates that this resulted in dust-related hazards not being fully controlled.

Deca-BDE was by far the most abundant PBDE component, but all PBDE exposures were significantly below nonbinding occupational exposure standards identified. This would imply that the risk of exposure to PBDEs from processing WUDS is low.

This study has highlighted that the processing of WUDS does not cause a high level of exposure to PBDEs; however, it has identified the need for improvement in the control of dust to reduce worker exposure to potentially hazardous bioaerosols.

## Supplementary Material

wxaf066_Supplementary_Data

## Data Availability

The data underlying this article will be shared on reasonable request to the corresponding author.

## References

[wxaf066-B1] ACGIH . no date. Endotoxins. October 2024. https://www.acgih.org/endotoxins/.

[wxaf066-B2] Altarawneh M, Saeed A, Al-Harahsheh M, Dlugogorski B. 2018. Thermal decomposition of brominated flame retardants (BFRs): products and mechanisms. Prog Energy Combust Sci. 70:212–259. 10.1016/j.pecs.2018.10.004.

[wxaf066-B3] Anon . 2019. Stockholm convention. http://www.pops.int. Accessed 19 August 2024.

[wxaf066-B4] ATSDR . 2017. Toxicological profile for polybrominated diphenyl ethers (PBDEs). https://www.atsdr.cdc.gov/toxprofiles/tp207.pdf. Accessed 19 August 2024. 37262200

[wxaf066-B5] Corry JEL, Curtis GDW, Baird RM. 2011. Handbook of culture media for food and water microbiology. RSC Publishing. 10.1039/9781847551450.

[wxaf066-B6] Duquenne P et al 2024. Bioaerosol exposure during sorting of municipal solid, commercial and industrial waste: concentration levels, size distribution, and biodiversity of airborne fungal. Atmosphere (Basel). 15:461. 10.3390/atmos15040461.

[wxaf066-B7] EA . 2022. Shredding waste upholstered domestic seating containing POPs: RPS 264. GOV.UK. https://www.gov.uk/government/publications/shredding-waste-upholstered-domestic-seating-containing-pops-rps-264/shredding-waste-upholstered-domestic-seating-containing-pops-rps-264. Accessed 19 August 2024.

[wxaf066-B8] EA . 2023. Segregating waste upholstered domestic seating that may contain persistent organic pollutants (POPs) at HWRCs: RPS 266. GOV.UK: EA. https://www.gov.uk/government/publications/segregating-waste-upholstered-domestic-seating-that-may-contain-pops-at-hwrcs-rps-266/segregating-waste-upholstered-domestic-seating-that-may-contain-persistent-organic-pollutants-pops-at-hwrcs-rps-266. Accessed 19 August 2024.

[wxaf066-B9] EFSA Panel on Contaminants in the Food Chain (CONTAM) et al 2024. Update of the risk assessment of polybrominated diphenyl ethers (PBDEs) in food. EFSA J. 22:e8497. 10.2903/j.efsa.2024.8497. 38269035 PMC10807361

[wxaf066-B10] Fernandes A, White S, D'Silva K, Rose M. 2004. Simultaneous determination of PCDDs, PCDFs, PCBs and PBDEs in food. Talanta. 63:1147–1155. 10.1016/j.talanta.2004.05.039. 18969544

[wxaf066-B11] Gravel S, Aubin S, Labrèche F. 2019. Assessment of occupational exposure to organic flame retardants: a systematic review. Ann Work Expo Health. 63:386–406. 10.1093/annweh/wxz012. 30852590

[wxaf066-B12] Hansen KK et al 2024. Microbial exposure during recycling of domestic waste: a cross-sectional study of composition and associations with inflammatory markers. Occup Environ Med. 81:580–587. 10.1136/oemed-2024-109628. 39557564 PMC11671902

[wxaf066-B13] Health Council of the Netherlands . 2010. Endotoxins. Health-based recommended occupational exposure limit. The Hague Health Council of the Netherlands. https://www.healthcouncil.nl/documents/advisory-reports/2010/07/15/endotoxins-health-based-recommended-occupational-exposure-limit. Accessed 19 August 2024.

[wxaf066-B14] Hebisch R, Linsel G. 2012. Workers´ exposure to hazardous substances and biological agents in recycling enterprises. Gefahrst Reinhalt Luft. 72:163–169.

[wxaf066-B15] HSE . 2013. The control of substances hazardous to health regulations 2002. Approved Code of Practice and guidance. (9780717665822). Bootle: HSE Books. https://www.hse.gov.uk/pubns/books/l5.htm. Accessed 19 August 2024.

[wxaf066-B16] HSE . 2014. General methods for sampling and gravimetric analysis of respirable, thoracic and inhalable aerosols. HSE. https://www.hse.gov.uk/pubns/mdhs/pdfs/mdhs14-4.pdf. Accessed 19 August 2024.

[wxaf066-B17] HSE . 2018. RR1126—In-cab Air filtration in plant vehicles to control exposure to hazardous dust: quarry industry example. HSE.

[wxaf066-B18] HSE . 2019. RR1151—Dust and bioaerosol exposure at municipal waste handling sites. https://webarchive.nationalarchives.gov.uk/ukgwa/20241207121227/https://www.hse.gov.uk/research/rrhtm/rr1151.htm. Accessed 19 August 2024.

[wxaf066-B19] HSE . 2022. Safety bulletin EPD1-2022. Ear loop respirators/masks do not provide protection as tight fitting RPE. https://www.hse.gov.uk/safetybulletins/ear-loop-respirators.htm. Accessed 19 August 2024.

[wxaf066-B20] IFA . no date. GESTIS substance database. https://gestis-database.dguv.de/. Accessed 19 August 2024.

[wxaf066-B21] Keeley-Lopez P, Turrell J, Peppicelli C, Vernon J. 2021. An assessment of persistent organic pollutants (POPs) in waste domestic seating. https://larac.org.uk/sites/default/files/10%20oCT Wk 3 WRc Final Report_UC15080.5_An assessment of persistent organic pollutants in waste domestic seating_270521.pdf. Accessed 19 August 2024.

[wxaf066-B22] Keen C, Sandys V, Crook B. 2024. Exposure to dust and bioaerosols at GB municipal waste handling sites. N Z J Health Safety Pract. 1:1–15. 10.26686/nzjhsp.v1i3.9624.

[wxaf066-B23] Madsen AM et al 2021. Review of biological risks associated with the collection of municipal wastes. Sci Total Environ. 791:148287. 10.1016/j.scitotenv.2021.148287. 34139489

[wxaf066-B24] Madsen AM, Rasmussen PU, Delsuz MS, Frederiksen MW. 2024. A cross-sectional study on occupational hygiene in biowaste plants. Ann Work Expo Health. 68:967–981. 10.1093/annweh/wxae074. 39312492 PMC11586275

[wxaf066-B25] Nandy S, Fortunato E, Martins R. 2022. Green economy and waste management: an inevitable plan for materials science. Prog Nat Sci-Mater Int. 32:1–9. 10.1016/j.pnsc.2022.01.001.

[wxaf066-B26] NIOSH . 2019. Dust control handbook for industrial minerals mining and processing. Second edition. By: Publication No. 2019–124, RI 9701. U.S. Department of Health and Human Services, Centers for Disease Control and Prevention, National Institute for Occupational Safety and Health, DHHS (NIOSH). Retrieved 23/06/2025. https://www.cdc.gov/niosh/docs/mining/UserFiles/works/pdfs/2019-124.pdf

[wxaf066-B27] SCOEL . 2012. Recommendation from the scientific committee on occupational exposure limits for diphenyl ether, octabromoderivative (commercial mixture). https://echa.europa.eu/documents/10162/35144386/109_diphenyl_ether_octabromoderivative_oel_en.pdf/ca32a60d-f97f-198a-14b6-abde752f8444?t=1691407253811. Accessed 19 August 2024.

[wxaf066-B28] Sjödin A, Patterson DG, Bergman Å. 2003. A review on human exposure to brominated flame retardants—particularly polybrominated diphenyl ethers. Environ Int. 29:829–839. 10.1016/S0160-4120(03)00108-9. 12850099

[wxaf066-B29] Statutory Instruments . 2007. The persistent organic pollutants regulations 2007; No. 3106. https://www.legislation.gov.uk/uksi/2007/3106/made#:%E2%88%BC:text=They%20enforce%20provisions%20relating%20to. Accessed 19 August 2024.

[wxaf066-B30] Swan J, Kelsey A, Crook B, Gilbert E. 2003. Occupational and environmental exposure to bioaerosols from composts and potential health effects—a critical review of published data. HSE. https://www.hse.gov.uk/research/rrhtm/rr130.htm. Accessed 19 August 2024.

[wxaf066-B31] TERA . no date. OARS WEEL Table. https://www.tera.org/OARS/PDF_documents/OARS_WEEL_Table.pdf. Accessed 19 August 2024.

[wxaf066-B32] UN . 2006. Stockholm convention persistent organic pollutants review committee meeting report UNEP/POPS/POPRC.2/17/Add.1 ‘Risk profile on commercial pentabromodiphenyl ether’. https://chm.pops.int/TheConvention/POPsReviewCommittee/Meetings/POPRC2/POPRC2ReportandDecisions/tabid/349/Default.aspx. Accessed 19 August 2024.

[wxaf066-B33] UN . 2007. Stockholm convention persistent organic pollutants review committee meeting report UNEP/POPS/POPRC.3/20/Add.6 ‘Risk profile on commercial octabromodiphenyl ether’. https://chm.pops.int/Default.aspx?tabid=348. Accessed 19 August 2024.

[wxaf066-B34] UN . 2014. Stockholm convention persistent organic pollutants review committee meeting report UNEP/POPS/POPRC.10/10/Add.2 ‘Risk profile on decabromodiphenyl ether (commercial mixture, c decaBDE)’. https://chm.pops.int/TheConvention/POPsReviewCommittee/Meetings/POPRC10/Overview/tabid/3779/mctl/ViewDetails/EventModID/871/EventID/514/xmid/11873/Default.aspx. Accessed 19 August 2024.

[wxaf066-B35] US-EPA . 2010. An exposure assessment of polybrominated diphenyl ethers (PBDE). https://cfpub.epa.gov/ncea/risk/recordisplay.cfm?deid=210404. Accessed 19 August 2024.

[wxaf066-B36] WHO . 2020. Food safety: persistent organic pollutants (POPs). WHO. https://www.who.int/news-room/questions-and-answers/item/food-safety-persistent-organic-pollutants-(pops). Accessed 19 August 2024.

[wxaf066-B37] WISH . 2023. Bioaerosols in waste and recycling. https://www.wishforum.org.uk/wp-content/uploads/2023/11/INFO-23-Bioaerosols-in-waste-and-recycling-V1-Oct-2023.pdf. Accessed 19 August 2024.

