# Supplementary material for: Worker exposure to persistent organic pollutants, as polybrominated diphenyl ethers, and biological hazards during the processing of waste upholstered domestic seating in Great Britain
Source: Ann Work Expo Health. 2025 Oct 23;70(1):wxaf066. doi: 10.1093/annweh/wxaf066 (PMC12821373; doi:10.1093/annweh/wxaf066)
Supplement: wxaf066_Supplementary_Data [file wxaf066_supplementary_data.pdf]

Worker exposure to persistent organic pollutants, as polybrominated diphenyl ethers, and biological hazards during the processing of waste upholstered domestic seating in Great Britain

Gosling, Rebecca J.[1]*, Simpson, Andrew T.[1], Bailey, Claire.[1], Baldwin, Peter EJ.[2], Lord, Samantha.[3]

[1] Health and Safety Executive (HSE), HSE Science and Research Centre, Harpur Hill, Buxton, Derbyshire SK17 9JN, UK

[2] Health and Safety Executive (HSE), Wellington Place, 2nd Floor, 7 & 8 Wellington Place, Leeds, West Yorkshire LS1 4AP, UK

[3] Health and Safety Executive (HSE), 100 Temple St, Redcliffe, Bristol BS1 6AG, UK

*Author to whom correspondence should be addressed. E-mail: Becky.Gosling@hse.gov.uk

© Crown copyright 2025

Supplementary Material

PDBE analysis method

For each air filter sample, the filter was removed from the cassette and placed into a clean extraction vessel. The internal walls of the cassette were swabbed three times with silanised glass wool soaked with hexane, and the swabs were placed in the same extraction vessel. The bulk dust samples were extracted in a mixture of hexane and acetone.

The content of the extraction vessel was fortified with a known amount of $^{13}C_{12}$ labelled PBDE internal standards ($^{13}C_{12}$ BDEs -28, -47, -99, -100, -153, -154, -183, -209). The fortified sample was left for an hour before being thrice extracted via sonification in dichloromethane:hexane (40:60 v/v) for 10 minutes. The combined extracts were then centrifuged and the supernatant concentrated to low volume. The concentrated supernatant was then treated with 37N sulphuric acid and mixed. The mixture was allowed to stand for 15 minutes to allow the aqueous acid and organic layers to separate. The aqueous layer was discarded and the acid treatment process was repeated on the remaining organic layer. The resulting organic extract was purified on two mini-columns in series, the upper column packed with acid modified silica gel on top of base modified silica, eluting directly onto the lower column containing activated alumina. The extract was fractionated through the mini-columns with hexane, before the lower column was then eluted with DCM:hexane (30:70 v/v). The eluates were combined and concentrated with the addition of a $^{13}C_{12}$ labelled internal sensitivity standard contained in nonane to approximately 50 µL. Extracts were analysed using high resolution gas chromatography coupled with high resolution (10k) mass spectrometry.

Quantification was performed using Isotope Dilution Mass Spectrometry which inherently includes recovery correction. Analytes which had no corresponding $^{13}C_{12}$ labelled internal standard were quantified against another of the internal standards of the same degree of bromination.

	LOD for air filter samples (ng/filter)	LOD for loose dust (µg/kg)
BDE-17	0.003	0.10
BDE-28/33	0.004	0.10
BDE-47	No data	0.10
BDE-49	0.004	0.10
BDE-66	0.008	0.10
BDE-71	0.004	0.10
BDE-77	0.006	0.10
BDE-85	0.004	0.10
BDE-99	0.024	0.10
BDE-100	0.004	0.10
BDE-119	0.006	0.10
BDE-126	0.004	0.10
BDE-153	0.014	0.10
BDE-138	0.016	0.10
BDE-154	0.007	0.10
BDE-183	0.010	0.10
BDE-209	0.748	200.00

Table S1. Sample analysis data from air samplers for each site visit

		Inhalable dust (mg/m³)	c-PentaBDE (mg/m³)	c-OctaBDE (mg/m³)	c-DecaBDE (mg/m³)	Bacteria at 25°C (CFU/m³)	Bacteria at 37°C (CFU/m³)	Actinomycetes (CFU/m³)	Lactase positive coliforms (CFU/m³)	Fungi at 25°C (CFU/m³)	Aspergillus fumigatus (CFU/m³)	Endotoxin (EU/m³ 8-hr TWA)
Site 1	Personal - Labourer	1.43	7.21E-08	8.77E-08	6.05E-05	1.31E+05	7.82E+04	3.31E+04	4.97E+03	6.21E+04	6.21E+03	2.43E+02
Site 1	Personal - Labourer	1.35	7.20E-08	8.76E-08	3.75E-05	7.93E+03	4.41E+03	2.50E+04	1.60E+02	6.28E+04	3.72E+03	7.30E+01
Site 1	Personal - Supervisor	0.16	7.19E-08	8.74E-08	1.15E-05	4.97E+03	2.95E+03	2.95E+03	<51.80	3.42E+04	<51.80	9.29E+00
Site 1	Personal - Driver	0.46	1.02E-07	8.94E-08	1.18E-05	6.51E+03	3.03E+03	4.63E+03	6.07E+02	3.86E+03	7.17E+02	1.50E+01
Site 1	Personal - Driver	0.15	7.51E-08	9.13E-08	1.20E-05	3.47E+04	9.71E+03	7.71E+03	1.06E+03	6.49E+03	7.39E+02	5.19E+01
Site 1	Static - near shredder	2.68	1.08E-07	8.79E-08	3.02E-05	1.18E+04	6.25E+03	2.41E+04	6.51E+01	5.47E+04	4.56E+03	na
Site 1	Static - by vehicle doorway	0.88	2.52E-07	8.77E-08	1.99E-05	1.03E+04	5.70E+03	4.69E+04	2.53E+02	5.83E+04	5.07E+03	na
Site 1	Static - near POPS loading	1.10	1.09E-07	8.80E-08	2.12E-05	2.13E+04	3.07E+03	6.08E+04	6.68E+02	8.56E+04	1.94E+03	na
Site 2	Personal - Driver in POPs area	0.22	4.18E-08	7.40E-08	1.34E-04	6.37E+04	2.22E+04	1.73E+03	1.86E+03	1.92E+04	9.90E+03	1.09E+03
Site 2	Personal - Driver in other areas	0.20	9.52E-09	7.59E-08	2.57E-05	1.00E+03	4.44E+02	1.90E+02	6.35E+01	1.21E+03	1.90E+02	nd
Site 2	Static - by the pre-processed POPs pile	0.62	4.45E-07	7.11E-08	4.68E-04	1.50E+06	4.30E+05	1.63E+04	3.72E+03	6.62E+04	1.15E+04	na

Site 2	Static - on boundary between POPs and other areas	0.55	2.45E-07	6.89E-08	2.68E-04	5.83E+05	8.85E+04	5.97E+03	4.92E+03	7.44E+04	1.47E+04	na
Site 2	Static - between doors near POPs loading	1.88	2.19E-06	3.21E-07	2.14E-03	5.38E+06	7.74E+05	1.41E+04	5.31E+04	6.19E+04	1.62E+04	na
Site 3	Personal - Driver loading lorries	0.56	5.71E-08	4.35E-08	3.73E-05	2.52E+04	4.18E+03	2.57E+03	2.22E+03	1.06E+05	6.55E+03	1.05E+04
Site 3	Personal - Driver loading shredder	0.36	1.31E-08	2.41E-08	1.61E-05	5.12E+03	4.29E+03	1.41E+03	1.76E+03	5.61E+04	4.39E+02	2.05E+04
Site 3	Personal - Assistant Supervisor & driver	0.93	3.64E-08	2.58E-08	4.12E-05	3.40E+03	2.78E+04	2.01E+03	5.98E+03	2.53E+05	1.34E+03	1.16E+04
Site 3	Static - by processed POPS	0.86	5.66E-08	6.97E-08	4.75E-05	1.75E+04	6.66E+03	4.28E+02	3.12E+03	5.87E+05	1.28E+04	na
Site 3	Static - by shredder	1.70	3.95E-08	3.91E-08	7.02E-05	4.95E+04	1.89E+04	1.38E+04	1.89E+04	2.38E+06	2.51E+03	na
Site 3	Static - inside vehicle doorway	0.51	3.45E-08	2.62E-08	1.56E-05	9.55E+03	2.77E+04	3.20E+03	3.14E+03	6.41E+05	9.85E+03	na
Site 3	Static -outside vehicle doorway	<0.04*	7.24E-08	2.58E-08	3.28E-06	1.24E+03	6.21E+01	<62.10	6.21E+01	2.73E+03	<62.10	na
Site 4	Personal - Forklift driver	4.52	4.64E-07	1.20E-07	8.24E-05	8.66E+07	1.76E+07	2.01E+05	8.11E+04	6.57E+04	5.13E+04	8.88E+03
Site 4	Personal - Marshalling & driver	2.82	7.09E-07	1.35E-07	nd	1.89E+07	2.08E+06	9.85E+04	7.70E+03	1.25E+04	1.04E+04	1.04E+04

Site 4	Personal - Baler operator	5.26	5.87E-07	1.37E-07	nd	6.94E+08	2.82E+07	3.42E+05	6.10E+05	7.48E+04	4.61E+04	9.48E+03
Site 4	Personal - 360 excavator operator	0.06	5.01E-07	1.30E-07	nd	4.42E+06	1.13E+05	8.55E+04	4.13E+04	8.84E+03	1.20E+04	1.27E+04
Site 4	Personal - Marshalling & driver	1.13*	5.96E-07	1.54E-07	2.30E-05	2.13E+07	6.53E+06	1.08E+05	1.18E+04	3.42E+04	7.34E+03	5.89E+03
Site 4	Static - inside vehicle doorway	1.46	5.75E-07	1.49E-07	9.60E-06	5.04E+05	3.10E+04	1.08E+05	1.38E+03	5.36E+04	2.84E+04	na
Site 4	Static - by baler	5.46	2.33E-06	1.60E-07		3.90E+07	2.33E+07	2.50E+05	1.13E+05	9.97E+04	6.23E+04	na
Site 4	Static - by shredder	4.04	6.25E-07	1.62E-07	4.68E-05	3.61E+07	1.01E+07	2.53E+05	1.45E+04	6.28E+04	4.47E+04	na
Site 5	Personal - 360 excavator driver	0.95	2.80E-06	1.00E-07	nd	1.49E+03	6.63E+03	5.20E+02	<65.0	5.78E+04	1.62E+03	6.50E-01
Site 5	Static - inside vehicle doorway	0.43	2.10E-06	2.40E-07	1.32E-05	1.01E+04	2.35E+04	2.41E+04	2.19E+02	2.19E+05	7.66E+04	na
Site 5	Static - outside vehicle doorway	0.05	2.20E-06	8.00E-08	5.33E-04	3.39E+02	6.10E+02	6.78E+01	<67.8	1.56E+03	6.78E+01	na
Site 5	Static - on shredder control panel next to tipping point	0.99	2.10E-06	7.80E-07	nd	1.11E+04	2.41E+04	7.91E+03	1.65E+02	2.69E+05	1.04E+05	na
Site 5	Static - on out of use conveyor to baling plant	0.36	2.10E-06	2.00E-07	nd	2.56E+03	1.43E+04	4.16E+03	5.34E+01	3.90E+04	5.34E+04	na
Site 5	Static - by processed POPs tipping point	0.58	2.10E-06	2.20E-07	nd	6.38E+03	2.08E+04	6.28E+03	1.56E+02	6.28E+04	6.74E+04	na

nd - no data, na - not applicable

Table S2. Summary of exposures and controls by site

	Site 1	Site 2	Site 3	Site 4	Site 5
Infrastructure	Mobile shredder, short open conveyor with one drop.	Mobile shredder, short open conveyor with one drop.	Fixed shredder with long open conveyor and metal sorter with three drops.	Fixed shredder with long open conveyor, baler/metal sorter and cross wrapper, with two drops.	Fixed shredder, long conveyor system, partially covered or with high sides, directed through a disused metal sorter, with three drops.
Shipments out	8-10 trailers of mixed waste.	3 trailers of processed WUDS &<br>8 other lorries of (unprocessed) municipal waste.	~12 trailers of mixed waste.	6 trailers of mixed waste as bales.	1 skip of processed WUDS.
8-hr TWA Inhalable dust exposures (mg/m³)	0.19<br>0.20<br>0.58<br>1.69<br>1.79	0.25<br>0.28	0.43<br>0.67<br>1.10	0.71<br>1.34‡<br>3.35<br>5.37<br>6.25	0.95
Static background air samples (mg/m³)	0.88<br>1.10<br>2.68	0.55<br>0.62<br>1.88	0.51<br>0.86<br>1.70	1.46<br>4.04<br>5.46	0.36<br>0.43<br>0.58<br>0.99
Segregation	Two workers in cabs with filtration.	Both workers in cabs with HEPA filtration.	All workers in cabs with HEPA filtration.	Two workers in cabs with filtration (one	Worker in cab with filtration.

				with open windows), & one in a cab without.	
LEV	None	None	None	None	None
Water misters/ suppression	Twelve roof level water mist fans above waste piles, shredding, and unloading/ loading areas.	Two water dust suppression cannons aimed at processed material. The water contained a binder.	None	Two high level water mist fans directed at unprocessed waste piles. Two others unused.	Roof level misters but not used.
Mechanical general ventilation	Six roof fans unused.	Six wall fans unused.	Large ducted extract ventilation system.	None	None
Natural ventilation	Six low level wall vents & four open doorways.	Twenty high level wall vents & six open doorways.	Two low level wall vents.	Four open doorways.	One open doorway.
RPE	FFP3 and P3 reusable masks available.	One worker wore a FFP3 mask outside of cab. PAPR was available.	Three workers wore FFP3 masks outside of cabs.	One worker wore a FFP3 mask all shift, and two wore FFP2 masks for cleaning.	None available.
Cleaning	Two labourers periodically with brushes and shovels.	No hands-on cleaning observed during the visit.	Three workers with scrapers, brushes and shovels over six sessions.	Five workers for ~2 hours at shift end.	Limited cleaning with a vacuum.

‡ Result considered unreliable due to presence of a large (1-2 mm) particle in the cassette and sample timer issues
